# Computational Model of Vascular Endothelial Growth Factor Spatial Distribution in Muscle and Pro-Angiogenic Cell Therapy

**DOI:** 10.1371/journal.pcbi.0020127

**Published:** 2006-09-22

**Authors:** Feilim Mac Gabhann, James W Ji, Aleksander S Popel

**Affiliations:** Department of Biomedical Engineering, Johns Hopkins University School of Medicine, Baltimore, Maryland, United States of America; Medical College of Wisconsin, United States of America

## Abstract

Members of the vascular endothelial growth factor (VEGF) family of proteins are critical regulators of angiogenesis. VEGF concentration gradients are important for activation and chemotactic guidance of capillary sprouting, but measurement of these gradients in vivo is not currently possible. We have constructed a biophysically and molecularly detailed computational model to study microenvironmental transport of two isoforms of VEGF in rat extensor digitorum longus skeletal muscle under in vivo conditions. Using parameters based on experimental measurements, the model includes: VEGF secretion from muscle fibers; binding to the extracellular matrix; binding to and activation of endothelial cell surface VEGF receptors; and internalization. For 2-D cross sections of tissue, we analyzed predicted VEGF distributions, gradients, and receptor binding. Significant VEGF gradients (up to 12% change in VEGF concentration over 10 **μ**m) were predicted in resting skeletal muscle with uniform VEGF secretion, due to non-uniform capillary distribution. These relative VEGF gradients were not sensitive to extracellular matrix composition, or to the overall VEGF expression level, but were dependent on VEGF receptor density and affinity, and internalization rate parameters. VEGF upregulation in a subset of fibers increased VEGF gradients, simulating transplantation of pro-angiogenic myoblasts, a possible therapy for ischemic diseases. The number and relative position of overexpressing fibers determined the VEGF gradients and distribution of VEGF receptor activation. With total VEGF expression level in the tissue unchanged, concentrating overexpression into a small number of adjacent fibers can increase the number of capillaries activated. The VEGF concentration gradients predicted for resting muscle (average 3% VEGF/10 μm) is sufficient for cellular sensing; the tip cell of a vessel sprout is approximately 50 **μ**m long. The VEGF gradients also result in heterogeneity in the activation of blood vessel VEGF receptors. This first model of VEGF tissue transport and heterogeneity provides a platform for the design and evaluation of therapeutic approaches.

## Synopsis

It is not currently possible to experimentally quantify the gradients of protein concentration in the extracellular space in vivo. However, the concentration gradients of vascular endothelial growth factor (VEGF) are essential for both initiation and directed guidance of new blood vessels. The authors develop a computational model of VEGF transport in tissue in vivo (skeletal muscle, though the method is applicable to other tissues and other proteins) with realistic geometry and including biophysical interactions of VEGF, its receptors, and the extracellular matrix. Using this model, the authors predict for the first time the distribution of VEGF concentration and VEGF receptor activation throughout the tissue. VEGF concentration gradients are significant, up to 12% change in VEGF concentration over 10 μm in resting muscle. Transplanting VEGF-overexpressing myocytes (for therapeutic induction of blood vessel growth) increases the gradients significantly. Endothelial cells in sprouting vessels are approximately 50 μm long, and therefore the predicted gradients across the cell are high and sufficient for chemotactic guidance of the new vessels. The VEGF concentration gradients also result in significant heterogeneity in the activation of VEGF receptors on blood vessels throughout the tissue, a possible reason for the sporadic nature of sprout initiation.

## Introduction

Vascular endothelial growth factor (VEGF) is a key promoter of angiogenesis in vivo and it increases proliferation and migration of endothelial cells cultured in vitro [1]. In rats, there are five main splice variants of VEGF, denoted 120, 144, 164, 188, and 205 (corresponding to the number of amino acids), and the 120 and 164 isoforms are the most prevalent [2]. VEGF is expressed at different levels (ranging over four orders of magnitude) by a variety of cells throughout the body including skeletal muscle [3–5]. VEGF_164_ is secreted as a 45-kDa homodimeric glycoprotein containing an exon-7 encoded domain which allows binding to heparin and neuropilin-1 (NRP-1). VEGF_120_ is also a homodimeric glycoprotein (36 kDa) but is missing the exon-7 encoded domain. Because of this domain, only VEGF_164_ can bind to the heparan sulfate proteoglycans (HSPG) present in high concentrations in the extracellular matrix (ECM) and basement membrane (BM) spaces, and the two splice variants are responsible for different signaling in both physiological and cancer angiogenesis [6,7]. Furthermore, due to the presence of high concentrations of HSPG in the BM that surrounds VEGF-secreting cells (such as skeletal muscle myocytes), a large amount of VEGF_164_ becomes bound and sequestered near sources of VEGF secretion, creating a steep VEGF gradient [8]. The cellular response to VEGF occurs when signaling is initiated by the binding of VEGF to its cell surface receptor tyrosine kinases, VEGFR1 and VEGFR2. VEGF is degraded after it is internalized by these two VEGF receptors. The receptors and their interactions with VEGF and with each other are discussed in depth in [9].

VEGF is involved in both physiological (e.g., during exercise and wound healing) and pathological angiogenesis (e.g., in ~60% of human tumors and in age-related macular degeneration). VEGF upregulation is necessary for physiological angiogenesis under conditions of hypoxia via oxygen-sensing mechanisms in the HIF-1 pathway [10] and increased shear stress in blood vessels [11]. In rat extensor digitorum longus (EDL) muscle during exercise, both hypoxia and increased shear stress induce angiogenesis through overexpression of VEGF by myocytes [12]. Because of the importance of VEGF, many clinical trials are under way for both pro- and anti-angiogenic therapies [13–15]. The FDA has approved anti-VEGF treatments including Avastin (bevacizumab), an antibody to VEGF, and Macugen (pegaptinib), an RNA aptamer which binds to and sequesters human VEGF_165_. Use of VEGF as a pro-angiogenic treatment for cardiac and limb ischemia has generated intense interest but direct administration of VEGF has not yet produced effective results in humans, and initial trials of transplantation of angiogenic cells have proven effective but require further work [16–18].

To create an effective VEGF-driven pro-angiogenic therapy, better understanding is needed of both physiological and pathological VEGF-induced angiogenesis. Increasing VEGF concentration leads to angiogenesis, but concentrations of VEGF beyond critical levels may result in formation of abnormal vessels (leaky, tumor-like, and with larger lumens) and hemangiomas [19,20]. Thus, normal blood vessels can only be formed if microenvironmental amounts of VEGF are carefully maintained above threshold levels for therapeutic angiogenesis but below threshold levels for abnormal angiogenesis over a prolonged period of time. Furthermore, responses to VEGF depend on not only VEGF concentration but also VEGF gradients, which enhance VEGF-induced angiogenesis and direct capillary growth [21–23]. Therefore, it is not sufficient to measure bulk quantities of VEGF in large samples of tissue in order to predict angiogenic behavior.

In the present study, we constructed a computational model to study extracellular diffusion of VEGF in vivo and effects of VEGF upregulation on gradients and receptor binding using the well-characterized rat EDL tissue as a sample environment. We have previously constructed models studying the kinetics of VEGF binding to receptors on cells in vitro and the effect of the presence of NRP-1 or placental growth factor [24,25]. We have also shown using Monte Carlo methods that despite very small concentrations of free VEGF, a continuum description in terms of VEGF concentrations is justified [26]. Diffusion of VEGF in vitro has also been studied using a computational model [21]. However, to our knowledge, this is the first model of VEGF diffusion in a geometrically complex in vivo environment and it includes: transport of the two most abundant VEGF isoforms through ECM and BM, HSPG binding kinetics, VEGF secretion, and VEGF receptor binding and internalization kinetics. NRP-1 was not included in this model because there are currently no available measurements of the amount of NRP-1 in skeletal muscle. Using this model, we predict VEGF distribution and analyze VEGF gradients at a resolution that is currently impossible to measure experimentally. Use of this model will aid in understanding mechanisms of physiological and therapeutic VEGF overexpression. This model is general and may be built upon in the future to include additional molecular species as new experimental data are emerging, e.g., neuropilin expression level in skeletal muscle; it can also be applied to specific drug interactions and other tissue types.

## Results

### Model Formulation

#### Model geometry.

**Figure 1:**
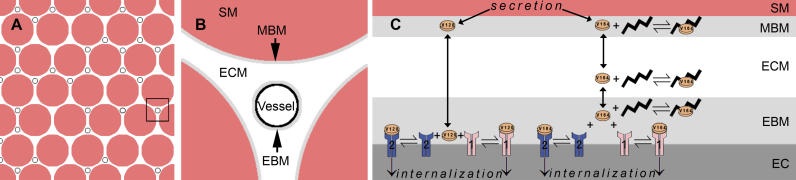
Schematics of VEGF Transport in Skeletal Muscle (A) Cross-sectional view of EDL tissue: red-filled circles represent muscle fibers, black unfilled circles represent capillaries located in the interstitium of the tissue. The fibers are assumed to be regularly spaced and hexagonally packed. (B) Interstitial space near a capillary: muscle fibers are surrounded by a thin MBM; capillaries formed by endothelial cells are surrounded by an EBM. The ECM lies in between the EBM and MBM, and VEGF diffuses throughout the interstitial space. (C) Diffusion and binding: two VEGF isoforms are secreted from the skeletal muscle myocyte into the MBM: VEGF_120_ and VEGF_164_ diffuse through the MBM, EBM, and ECM, but only VEGF_164_ is able to bind with HSPG in each layer. Near the endothelial cell surface (located in the EBM), VEGF can interact with VEGFR1 and VEGFR2, and both can be internalized whether bound to VEGF or unbound.

Rat EDL muscle geometry has been well-characterized physiologically and in its responses to hypoxia, hemodynamic shear stress, and electrical stimulation [11,27]. We have previously published a study of oxygen distribution in EDL muscle as related to angiogenesis [28], and the present study follows a similar methodology of geometric representation with several notable differences. A 2-D cross section of muscle with an area of 200 × 208 μm^2^ is represented by regions of muscle fibers and capillaries separated by interstitial space (Figure 1). At the edges of the tissue area, periodic boundary conditions for the molecular species under consideration are applied, as this is a small piece of tissue surrounded by the rest of the muscle. The dimensions of the area were chosen so that no geometric discontinuities would exist under these boundary conditions. Muscle fibers are explicitly represented in the present model as a separate phase from the interstitial space and thus are boundaries for VEGF transport. Based on photomicrographs [29] and interstitial volume tracer experiments for rat skeletal muscle [30], skeletal muscle fibers were represented by circles for simplicity, and they were staggered and packed in a uniform fashion (Figure 1A). Each fiber has a diameter of 37.5 μm for a cross-sectional area of 1,100 μm^2^, consistent with experimental measurements [31]. A total of 30 fibers were packed into the cross section accounting for 79.6% of total cross-sectional area.

Capillaries of 6 μm in external diameter and 0.5 μm wall thickness (5 μm in lumen diameter) were added randomly into the interstitial space between the muscle fibers (Figure 1B). The endothelial cells that line these vessels express VEGFR1 and VEGFR2 uniformly on their abluminal surface. The placement of capillaries was constrained by a minimum capillary-to-capillary distance parameter of 10 μm (measured from the centers of the lumens) and a minimum capillary-to-muscle-fiber distance of 1 μm (measured from the outer edge of a capillary to the outer edge of a muscle fiber). A total of 33 capillaries were placed randomly in the cross section. By measuring the distance from random points in the tissue to the center of the nearest capillary, we noted that our network closely matches experimentally measured vascular distribution [32] (average distance 15.5 μm (our model) versus 15.9 μm (experimental); 95th percentile of distance 29.8 μm versus 28.6 μm). The geometrical model is consistent with experimental observations of 800/mm^2^ capillary density in resting EDL [33]. The capillaries accounted for 2.2% of the cross-sectional area, and the interstitial space totals 18.1% of the cross-sectional area. All results presented here are for the same arrangement of capillaries, except where a homogenous capillary distribution is used and is so noted.

Each muscle fiber in the model is surrounded by a uniform thin myocyte basement membrane (MBM) layer (Figure 1B). Each capillary is surrounded by a uniform endothelial basement membrane (EBM) layer. The two BM spaces are separated by the ECM. The EBM and MBM each have distinct thickness based on electron microscope measurements (84.8 nm and 31.7 nm, respectively [34]) and have lower diffusivity and higher concentrations of HSPG than the ECM. Together, the BM and ECM components form the interstitial space within which VEGF diffuses.

Different units can be used to define each of the above parameters; the units given in the glossary (Table 2) are consistent with the equations presented below. The parameters may be converted to other units for comparison with appropriate experimental data, for example, molecules/cell or pmol/(μm^3^ tissue). These conversions are achieved by using the surface-area-to-tissue-volume ratios for myocytes (850 cm^2^/cm^3^) or blood vessels (150 cm^2^/cm^3^) in this tissue, and the area of the cell (1,000 μm^2^/endothelial cell; myocytes are discussed later).

**Table 2:**
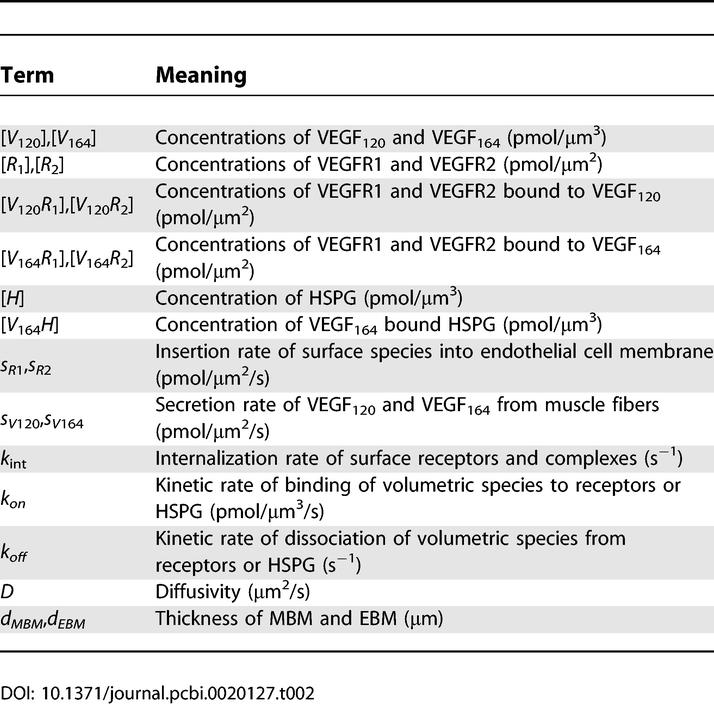
Glossary

#### Transport calculations and reaction kinetics.

In BM spaces, the membrane thickness is no more than 85 nm, and gradients across this distance are expected to be negligible, therefore free VEGF concentration is assumed to be uniform perpendicular to the capillary surface in the BM. Thus, at endothelial cell surfaces, the free VEGF concentrations are equal to those of its adjacent EBM spaces. VEGF that binds to receptors comes from the adjacent EBM, and VEGF that dissociates from receptors is released into the EBM; VEGF expressed by myocytes is secreted into an adjacent MBM space from which diffusion can then occur.

Transport within the ECM is described by mass balance equations:

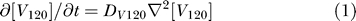


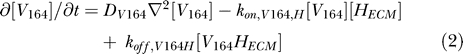
The mass balance equations describing VEGF transport between ECM and MBM are:

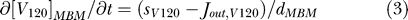


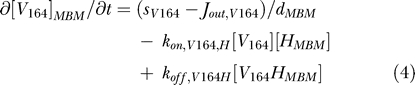
Here *d_MBM_* is the thickness of the MBM and *J_out_* is the Fickian diffusive flux of VEGF from the BM to the ECM. At steady state, the secretion of VEGF into the MBM and the diffusive flux of VEGF from BM to ECM are in equilibrium, but during transients *s_V120_* and *J_out_* have different values.

The mass balance equations describing VEGF transport between ECM and EBM are:

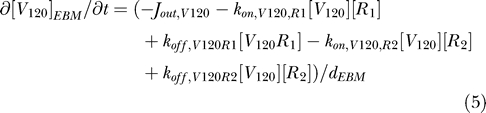


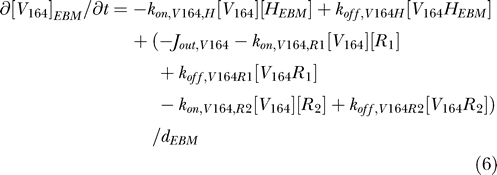
Here, *d_EBM_* is the thickness of the EBM.

On the endothelial cell surface, the binding kinetics between VEGF and receptors follows our previous study [24] in which VEGF can be bound to receptors and both free and bound receptors can be internalized. However, NRP-1 was not included in this model because it has not been quantified in skeletal muscle. Equations 7–12 apply at the surface of endothelial cells only because muscle fibers are assumed to express negligible amounts of VEGF receptors. The VEGF concentrations in Equations 7–12 are the concentrations in the EBM.

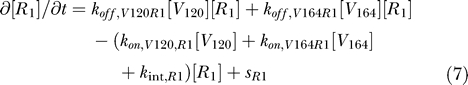


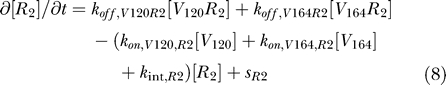


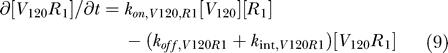


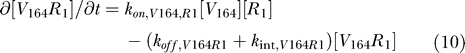


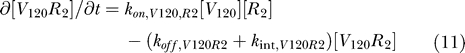


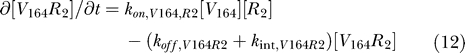
Binding kinetics between VEGF and HSPG in the interstitium is expressed as follows:

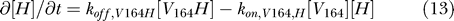


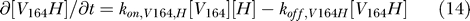
Here, [*H*] represents [*H_ECM_*], [*H_MBM_*], and [*H_EBM_*]; [*V*_164_ *H*] represents [*V*_164_ *H_ECM_*], [*V*_164_ *H_MBM_*], and [*V*_164_,*H_EBM_*] for binding reactions in the ECM, MBM, and EBM, respectively.

Thus, Equations 1, 2, 13, and 14 govern the concentration of VEGF in the ECM, while Equations 3–6 and 7–12 together form the boundary conditions at the myocyte cell surface (Equations 3–4) and endothelial cell surface (Equations 5–12). The notation in equations 1–14 is defined in Table 2.

### Model Parameters

The physiological parameters used in this model are summarized in Table 1. Experimentally measured parameters for VEGF transport in rat EDL are limited, and many parameters were estimated from experiments performed on rats and other species.

**Table 1:**
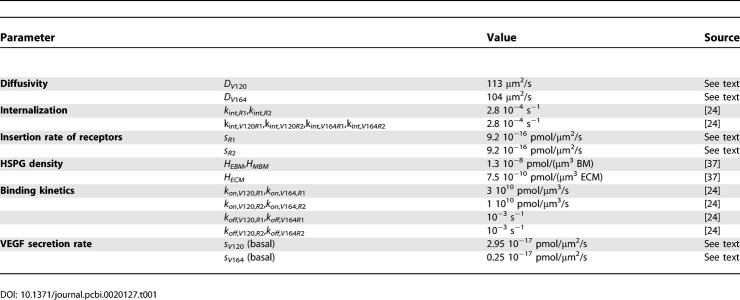
Parameters for VEGF Transport and Binding

#### In vivo diffusivity calculations.

VEGF diffusivity was calculated using a molecular-weight–based relationship for globular proteins [35] and adjusted to 37 °C using the Stokes–Einstein relation. We assume VEGF_120_ and VEGF_164_ aqueous diffusivities of 142 and 133 μm^2^/s. In skeletal muscle ECM, VEGF diffusion is hindered by collagen fibers (concentration: 75 mg/g ECM, fiber radius: 20 nm, fiber volume fraction: 0.14) and glycosaminoglycan chains (concentration: 5 mg/g ECM, fiber radius: 0.55 nm, fiber volume fraction: 7.8 10^−4^) [36]. To obtain in vivo values of diffusivity, we followed a method previously outlined [37] to calculate diffusive hindrance in interstitium. This predicts values of 113 and 104 μm^2^/s for VEGF_120_ and VEGF_164_ in vivo diffusivities, respectively.

#### HSPG and VEGF receptor density and kinetics.

HSPG concentration in ECM and BM spaces were obtained from values measured in human myocardium [37]. Currently, no in vivo measurements are available for the HSPG population in skeletal muscle. Effective VEGF–HSPG binding kinetics in vivo has not been measured, so we use those determined for the fibroblast growth factor (FGF-2). In vitro studies confirm that binding kinetics is similar for reactions of FGF-2 and VEGF binding to HSPG [38].

VEGF receptor concentrations and kinetic rates were chosen based on in vivo measurements of total VEGFR2 protein concentration and capillary density in human vastus lateralis skeletal muscle [39,40]. For that tissue, total protein concentration in skeletal muscle is assumed to be 150 mg/g muscle [41], endothelial cell surface area is 1,000 μm^2^, and capillary diameter is 7 μm. The in vivo measurements correspond to an average VEGFR2 density of 20,000 receptors per cell, which is of the same order of magnitude as previously measured in vitro values of 50,000 [24]. However, no study has determined the percentage of total VEGF receptors expressed on the abluminal capillary surface, within the cell, and on the luminal cell surface. In this study, we assume as a baseline that 50% of total receptors (10,000 VEGFR2/cell) is expressed on the abluminal cell surface (available for VEGF binding), and we perform a sensitivity analysis to study the effects of expressing more or fewer receptors.

In vivo measurements in human show that approximately ten times as much VEGFR1 is expressed as VEGFR2 [39,40]. However, soluble VEGFR1 (sVEGFR1) is also present in interstitial spaces of skeletal muscle (not included in this model), and no studies in skeletal muscle have reported the ratio of VEGFR1 to sVEGFR1. In our study, we assume that an equal amount of VEGFR1 is expressed on the abluminal extracellular surface as VEGFR2, and we have also performed sensitivity analyses to study the effect of changing VEGFR1 and VEGFR2 cell surface concentrations on the formation of VEGF gradients. VEGF receptor kinetic rates obtained from in vitro binding studies [24] were assumed to hold in vivo*.*

#### Estimates of VEGF expression.

Currently, only in vitro measurements of VEGF protein secretion have been published [3,42]. In addition, measurements of total VEGF in rat includes VEGF located intracellularly [11] and bound VEGF, which is sensitive to HSPG and sVEGFR1 concentrations. To simulate realistic in vivo conditions, expression rates for VEGF_120_ and VEGF_164_ were obtained by adjusting values so that our model matches experimental in vivo measurements of unbound VEGF in human skeletal muscle [43]. Under pre- and post-exercise resting conditions, unbound VEGF concentrations range between 0.6 and 1.5 pM, so in our study we target a free concentration of approximately 1 pM under resting conditions. Under basal conditions (to meet the requirement of 1 pM free VEGF concentration), muscle fibers express VEGF at a rate of 3.2 10^−17^ pmol/μm^2^/s (2.7 fmol/(L tissue)/s), though different receptor densities would require different secretion rates to achieve the same protein concentration.

Skeletal myocytes are multinucleate cells, hence they cannot be directly compared with the mononucleate cells used to measure VEGF secretion in vitro experimentally. The myonuclear domain (MD) is the volume and associated cell membrane surface area corresponding to (and under the control of) each nucleus of the cell. To make a valid comparison, secretion from this surface area is assumed to be under the control of a single nucleus. By counting the number of nuclei per unit length of the fiber, and the cross-sectional area, the fiber surface area of each MD was shown to decrease with the cross-sectional area of the fiber [44]. For a cylindrical fiber with a diameter of 37.5 μm, the predicted MD surface area in rat muscle is approximately 2500 μm^2^. The VEGF secretion rate used in our model corresponds to 0.048 molecules/MD/s and secretion from each MD can be compared with that from a mononucleate cell. The secretion rate in our model is comparable to in vitro measurements in various tissues including rat skeletal muscle: 0.01 in rat tibialis anterior [45], 0.08 in rat adipocyte [46], and 0.10 in rat cavernous smooth muscle [47] (all in molecules/cell/s). Studies have demonstrated that VEGF secretion rates range over four orders of magnitude between normal and tumor tissue (0.001 versus 11.6 molecules/cell/s) [3,5]. Furthermore, cell-based pro-angiogenic therapies have been studied using transgenic myoblasts expressing VEGF at levels between 20-fold and 200-fold basal (0.85 to 9.3 molecules/cell/s for different transgenic clones) [19,20].

Using relative mRNA abundances for splice variants of VEGF in mouse skeletal muscle [2], assuming a linear relationship between VEGF protein secretion and mRNA levels [10] and only these two isoforms present, VEGF_164_ secretion is nearly 12 times higher than VEGF_120_ secretion (2.95 versus 0.25 10^−17^ pmol/μm^2^/s, or 0.044 versus 0.004 molecules/MD/s). Both isoforms are secreted by the myocytes.

### Results of Computer Simulations

#### Basal conditions—Uniform VEGF secretion.

**Figure 2:**
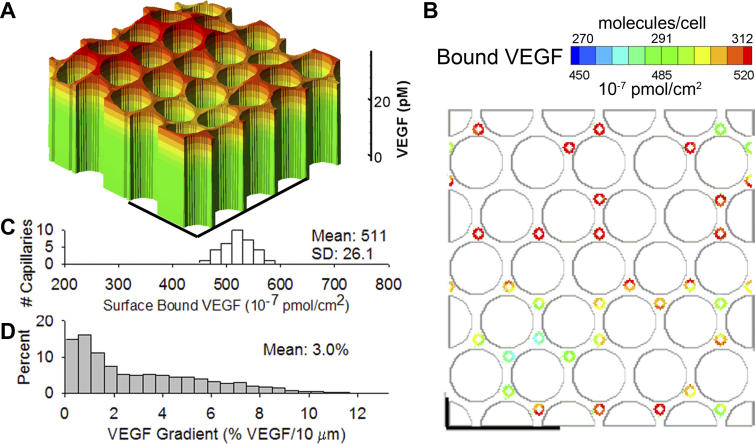
VEGF Distribution in Resting Skeletal Muscle for Uniform Secretion of VEGF (A) VEGF concentration variations in skeletal muscle. The surface represents the total VEGF concentration (free plus HSPG-bound) across the interstitial space. (B) Graphical representation of VEGF binding: large gray circles represent muscle fibers. Small circles represent capillaries and are color-coded to show the amount of VEGF bound to the surface of the capillary. (C) Histogram of average VEGF binding to capillaries. Each capillary has a different amount of bound VEGF due to the spatial variations of VEGF. Some capillaries may be activated while others are not. (D) Histogram of VEGF gradients. The percentage of tissue that experiences VEGF gradient of a certain magnitude. Gradient is defined as the change in VEGF concentration over 10 μm divided by the mean VEGF concentration in the tissue. Capillary distribution in subsequent figures (except Figure 3A) is the same as Figure 2.

Under resting physiological conditions, each skeletal muscle fiber is assumed to secrete VEGF at the same rate because all muscle fibers are well-oxygenated. The uniform secretion simulation results are summarized in Figure 2. The total VEGF content in muscle (free, bound to HSPG, and bound to cell surface receptors; not including intracellular VEGF, e.g., inside muscle fibers) is predicted to be 693 pg/(cm^3^ tissue). Of total VEGF, 50% is bound to endothelial cell receptors (VEGFR1: 37%, VEGFR2: 13%), 49% is bound to HSPG (ECM: 36%, MBM: 9%, EBM: 4%), and 1% exists as freely diffusible VEGF. Of free VEGF, 7% is VEGF_120_ and the other 93% is VEGF_164_. The concentration of total (bound and free) VEGF is 17.5-fold higher in the MBM and 16.6-fold higher in the EBM compared with the ECM due to the higher concentration of HSPG in BM. On the endothelial cells, 2.3% of VEGFR1 and 0.8% of VEGFR2 is occupied by VEGF, corresponding to approximately 230 and 80 molecules bound per cell, respectively.

**Figure 3:**
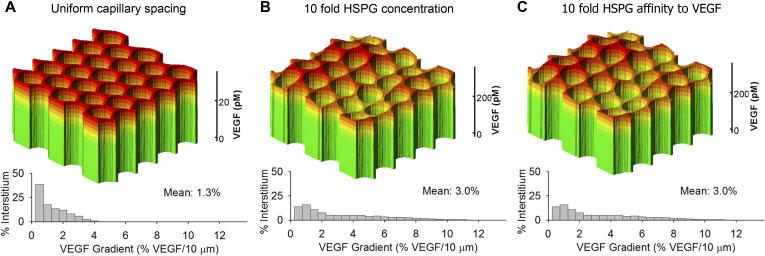
Effect of VEGF Capillary Distribution and HSPG on VEGF Gradients (A) Uniform capillary distribution results in a decrease in the average VEGF gradients in the tissue. (B,C) VEGF gradients at steady state are invariant (in percentage terms) to changes in the extracellular matrix composition. Increased density or affinity of VEGF binding sites results in increased bound VEGF content (and thus increased absolute values of the gradient), but no change in the relative gradient.

Under conditions of uniform secretion, relative VEGF gradients average 3.0% VEGF/10 μm and reach a maximum of 12.2% VEGF/10 μm in the ECM (relative gradients are measured as change in VEGF concentration across 10 μm, divided by mean VEGF tissue concentration); the scale of 10 μm is chosen as relevant to endothelial cell sensing of chemotactic gradients during sprout formation. VEGF concentrations and an analysis of gradients are visualized in Figure 2A. The most significant gradients exist between interstitial space with a high local capillary density and an area with low density, indicating that existence of VEGF gradients is dependent on heterogeneous placement of capillaries. When the capillaries are spaced homogeneously (30 capillaries are required for this), VEGF gradients decrease to an average of 1.3% VEGF/10 μm and a maximum of 3.7% VEGF/10 μm (Figure 3A).

#### Effect of VEGF receptor and HSPG concentration on VEGF gradient formation.

When HSPG binding affinity was increased to 10-fold basal value (by increasing *k_on_*,*_V_*_164,*H*_), or the concentrations of HSPG in ECM, EBM, and MBM were all increased 10-fold, the total VEGF concentration in interstitial space increased 10-fold but surface receptor binding and relative VEGF gradients were unaffected (average of 3% VEGF/10 μm) (Figure 3B and 3C). Absolute values of concentration gradients (e.g., in pM per μm) would obviously be increased due to the increased average concentration. However, at steady state the HSPG in the matrix does not affect the relative gradient.

**Figure 4:**
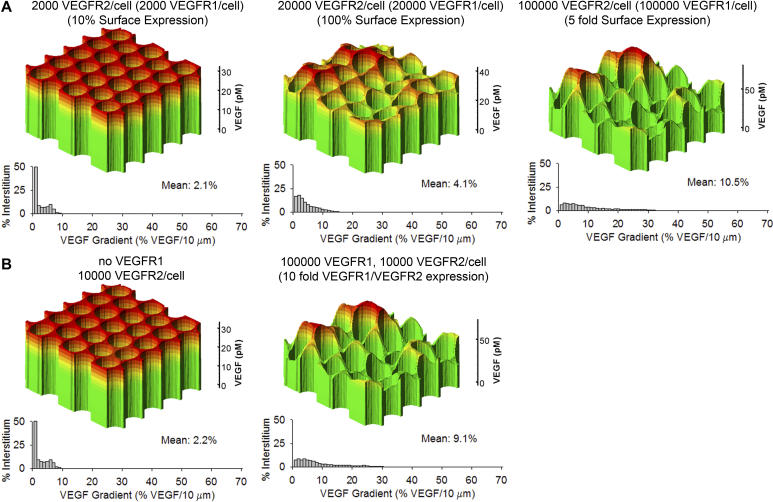
VEGF Receptor Density Alters Magnitude of VEGF Gradients Increasing VEGFR2 density (A) and increasing VEGFR1 density (B) increase the gradients of VEGF concentration in tissue. Surfaces represent concentration of total VEGF (pM, free plus HSPG-bound) in the ECM across the cross section of tissue. Histograms of VEGF gradients and the percentage of tissue involved. Gradient defined as in Figure 2.

To study the effect of changing VEGF receptor concentrations on VEGF gradient formation, VEGF secretion levels were changed to maintain a mean unbound VEGF concentration of 1 pM. Decreasing VEGFR1 and VEGFR2 insertion rates to 20% each effectively decreases cell surface receptor concentrations to approximately 2,000 receptors/cell each (Figure 4A). This decreases VEGF gradients to an average of 2% VEGF/10 μm and also shifts the majority of VEGF from the cell surface to the interstitial space (from 49% to 88% of total VEGF; at 20% VEGF secretion). Doubling each VEGF receptor insertion rate corresponds to all VEGF receptors being expressed on the abluminal surface of endothelial cells (20,000 receptors/cell each). This nearly doubles maximum gradient (22.0% VEGF/10 μm) and increases the average gradient to 4.1% VEGF/10 μm (at 2-fold VEGF secretion). When VEGF receptor insertion rate is increased 10-fold to represent tissues with 10-fold receptor expression compared with the standard parameters (100,000 receptors/cell for VEGFR1 and VEGFR2), maximum VEGF gradient increases nearly 5-fold (63.4% VEGF/10 μm) and average gradient increases to 10.5% VEGF/10 μm (at 10-fold VEGF secretion).

Altering the ratio of VEGFR1 to VEGFR2 cell surface insertion rates follows the same trend (Figure 4B). If no VEGFR1 is expressed on the cell surface, VEGF gradients reduce to an average of 2.2% VEGF/10 μm (at 26% VEGF secretion). Conversely, increasing VEGFR1 cell surface expression 10-fold (representing a 10-to-1 cell surface expression of VEGFR1; 100,000 VEGFR1 and 10,000 VEGFR2/cell) increases gradients to 9.1% VEGF/10 μm on average (at 8-fold VEGF secretion). Overall, increasing VEGF receptor expression will increase VEGF gradients and require higher total VEGF secretion rates to maintain constant VEGF content in the tissue.

#### Binding and internalization kinetics sensitivity analysis.

**Figure 5:**
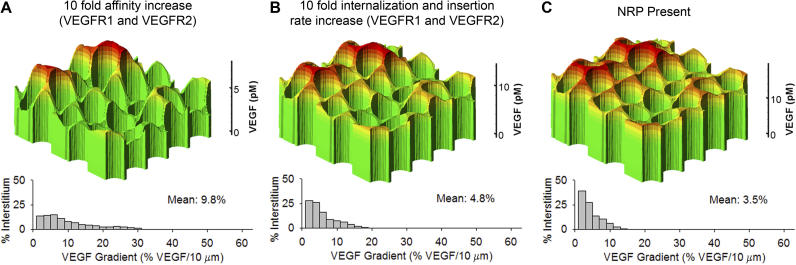
Effect of VEGF Receptor Kinetics on VEGF Gradients (A) Increased binding affinity (increased on-rate) has a similar outcome to increases in the receptor density: the VEGF gradients are magnified. (B) Increased internalization rate of VEGF-receptor complexes results in an increase in the VEGF gradients.

When VEGF receptor affinity is increased 10-fold, VEGF gradients increase to an average of 9.8% VEGF/10 μm and a maximum of 60.1% VEGF/10 μm (Figure 5A). The change in affinity reduces the amount of interstitial VEGF required to achieve the same quantity of VEGF bound on the cell surface and the same rate of VEGF internalization. Increasing VEGF receptor internalization rate 10-fold similarly reduces the amount of bound VEGF (and therefore free VEGF) required to achieve the same level of total VEGF internalization. Total VEGF receptor concentrations (free and bound) are maintained constant by increasing receptor insertion rates. Gradients increased to an average of 4.8% VEGF/10 μm and a maximum of 25.4% VEGF/10 μm (Figure 5B). Our study shows that VEGF gradients are highly dependent on cell surface receptor binding affinity and internalization rates for which there are no direct in vivo measurements available. However, the trends show that increasing either receptor binding affinity or internalization rate (while maintaining receptor density) will increase VEGF gradients and reduce the interstitial level of VEGF necessary to achieve the same quantity of bound VEGF. In skeletal muscle, NRP-1 expression has not been measured, and therefore we have not included it explicitly. The presence of NRP-1 would effectively increase the affinity of VEGFR2 and decrease the affinity of VEGFR1 for VEGF_164_, so its net effect on VEGF gradients depends on the relative expression of the two receptors.

#### Uniform VEGF upregulation.

VEGF expression is upregulated 4-fold in exercise [39] and 6-fold in chronic electrical stimulation [10] protocols. Uniformly increasing VEGF secretion rates 10-fold in all muscle fibers in the simulations results in a linear response in increases of both free and bound VEGF concentration (Figure S1). Between basal secretion and 10-fold VEGF overexpression, total VEGF concentration increased from 0.7 to 7.6 ng/(cm^3^ tissue) (11-fold increase). The fractional occupancy of VEGFR1 increased 9.6-fold (2.3% to 22.1%) and that of VEGFR2 increased 12-fold (0.8% to 9.6%). Free and bound VEGF in the interstitium increase proportionally, so absolute VEGF gradients (change in VEGF concentration over a distance) also increase proportionally. Relative VEGF gradients (i.e., gradients normalized by mean VEGF concentration in the interstitial space), are the same as the gradients under basal conditions (average of 3% and maximum of 12% VEGF/10 μm) (Figure S2).

Under uniform secretion, the bound VEGF concentration on capillaries fits an approximately normal distribution with a mean and standard deviation of 511 and 26.1 10^−7^ pmol/cm^2^, respectively (Figure 2C). When VEGF is uniformly overexpressed, the bound VEGF concentrations increase and continue to fit a tight normal distribution as in basal conditions with a mean of 5112 and standard deviation of 194 10^−7^ pmol/cm^2^.

#### Single and multiple fiber upregulation.

**Figure 6:**
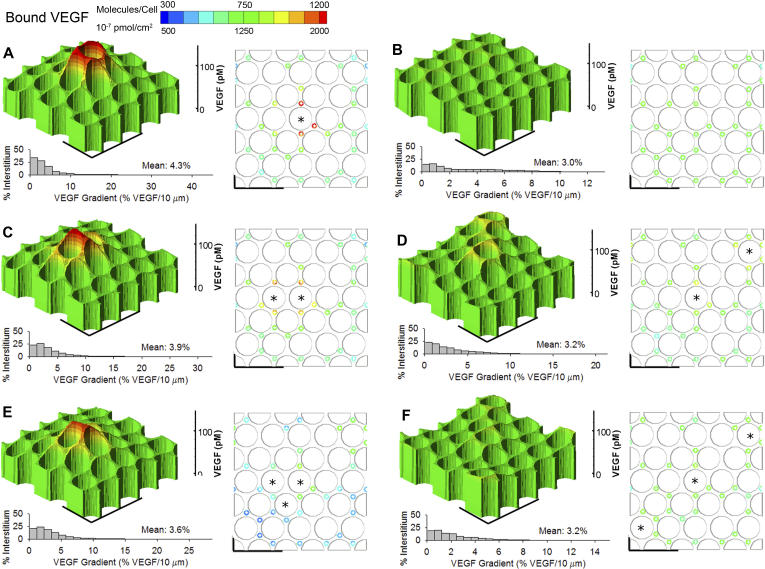
Cell-Based Delivery of VEGF to Muscle The total VEGF expression in each of the tissues (A–F) is the same; the arrangement delivery of VEGF from cells incorporated into each tissue is different. Stars mark the fiber(s) that overexpress VEGF. VEGF gradients and capillary activation graphs are as for Figure 2. (A) 40-Fold overexpression of VEGF in one fiber. (B) Uniform 1.33-fold overexpression of VEGF by all fibers. (C,D) 20-Fold overexpression of VEGF in two fibers, close together (C) or distant (D). (E,F) 13.3-Fold overexpression of VEGF in three fibers, close together (E) or distant (F).

To predict the impact of cell-based therapy in delivering VEGF to muscle, we examined the effects of overexpression in one fiber versus distributed overexpression. Specific muscle fibers were chosen to chronically overexpress VEGF such that the number and spatial positions of overexpressing fibers varied while total secretion in the tissue was kept constant. Six conditions were studied: single fiber at 40-fold overexpression, two adjacent fibers at 20-fold, two distant fibers at 20-fold, three adjacent fibers at 13.3-fold, three distant fibers at 13.3-fold, and uniform secretion at 1.3-fold above basal level (while remaining fibers secrete at basal level), and the results are summarized in Figure 6.

Total VEGF increases equally in all cases but much larger gradients are generated by local overexpression. Under single-fiber 40-fold overexpression (Figure 6A), VEGF gradients average 4.3% VEGF/10 μm and reach a maximum of 44.1%/10 μm (43% and 261% higher than uniform expression, respectively). For overexpression in two fibers at 20-fold over basal level (Figure 6C and 6D), VEGF gradients are sensitive to the spatial distribution of overexpressing fibers. Adjacently placed overexpressing fibers produce VEGF gradients averaging 3.9% VEGF/10 μm reaching a maximum of 33.1% VEGF/10 μm, while distantly placed fibers (106 μm between the centers of the fibers) average 3.2% VEGF/10 μm and reach a maximum of 20.6% VEGF/10 μm. For three-fiber overexpression at 13.3-fold over basal level (Figure 6E and 6F), the results follow the trend of two-fiber overexpression. Three adjacent overexpressing fibers produce gradients averaging 3.6% VEGF/10 μm at a maximum of 28.3% VEGF/10 μm, while three distant overexpressing fibers (106 μm between the centers of the fibers) produce gradients averaging 3.2% VEGF/10 μm at a maximum of 14.2% VEGF/10 μm.

**Figure 7:**
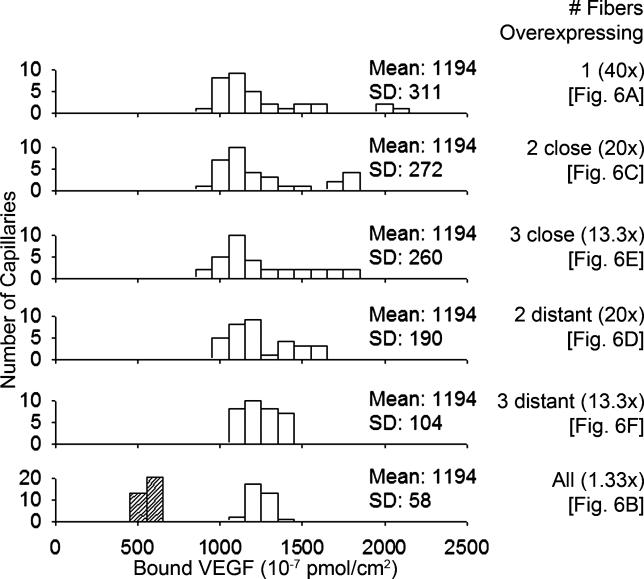
VEGF Binding to Capillaries for Cell-Based Delivery of VEGF to Muscle Each vessel in each tissue experiences a different level of VEGF binding. Total VEGF expression level is the same in each tissue, and the mean VEGF binding to capillaries is the same, but the concentrating of VEGF overexpression into a small number of adjacent fibers results in increased variability of binding. The tissues are arranged in decreasing order of standard deviation of VEGF-capillary binding, as a metric of the variability in capillary activation within the tissue. The more concentrated the VEGF overexpression, the higher the variability. Each tissue is labeled with the number of fibers overexpressing VEGF, the level of overexpression in each fiber, and the panel in Figure 6 for the corresponding VEGF concentration and receptor activation. The shaded bars in the bottom graph represent VEGF binding to capillaries under basal (no overexpression) conditions.

The differences in VEGF gradients for the overexpression models directly affect cell surface receptor VEGF binding. For single-fiber overexpression (Figure 6A, Figure 7), the three capillaries adjacent to the overexpressing fiber bind up to 2,050 10^−7^ pmol/cm^2^ of VEGF while all other capillaries bind a maximum of 1,609 10^−7^ pmol/cm^2^ (27% difference). Capillaries over 65 μm from the center of the overexpressing fiber (20 capillaries) bind a maximum of 1,242 10^−7^ pmol/cm^2^ (65% difference). For overexpression in two or three fibers, adjacent overexpression produces larger heterogeneities in capillary VEGF binding (standard deviation: 272 and 260 versus 190 and 104 10^−7^ pmol/cm^2^, respectively) and higher maximum capillary bound VEGF (1,790 and 1,761 versus 1,577 and 1,385 10^−7^ pmol/cm^2^, respectively) and overexpression in three distant fibers produces maximum capillary VEGF binding near uniform levels (1,385 versus 1,342 10^−7^ pmol/cm^2^).

Overexpression from a single fiber produces a distinct threshold of VEGF binding between capillaries adjacent to overexpressing fibers and non-adjacent capillaries (Figure 7). As the number of overexpressing fibers is increased while total secretion is held constant, the distinction between VEGF bound to adjacent and non-adjacent capillaries becomes less apparent (Figure 7), and spatial distribution of overexpressing fibers affects VEGF gradients and greater distances between overexpressing fibers produces a far more uniform response with smaller gradients (Figure 6).

#### Effect of overexpression density—Increasing total VEGF secretion.

**Figure 8:**
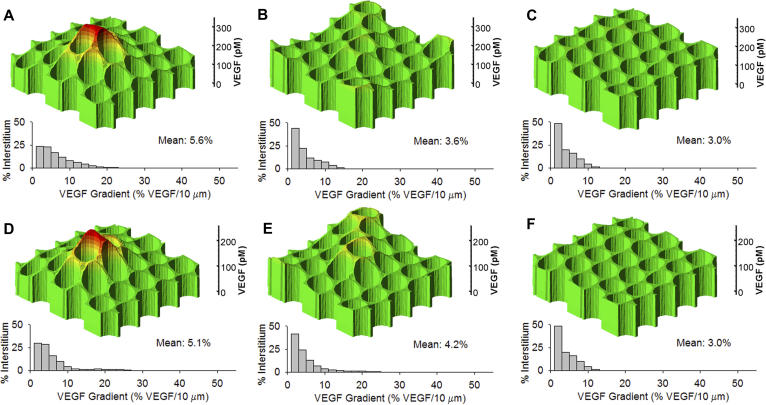
Increasing Dose of Cell-Based VEGF Delivery The delivery of two or three fibers overexpressing VEGF 40-fold is compared with an equivalent uniform overexpression. (A,B) VEGF concentration and gradients for three 40-fold overexpressing fibers close together (A) or distant (B). (C) Uniform VEGF overexpression of 4-fold. (D,E) VEGF concentration and gradients for two 40-fold overexpressing fibers close together (D) or distant (E). (F) Uniform VEGF overexpression of 2.67-fold.

**Figure 9:**
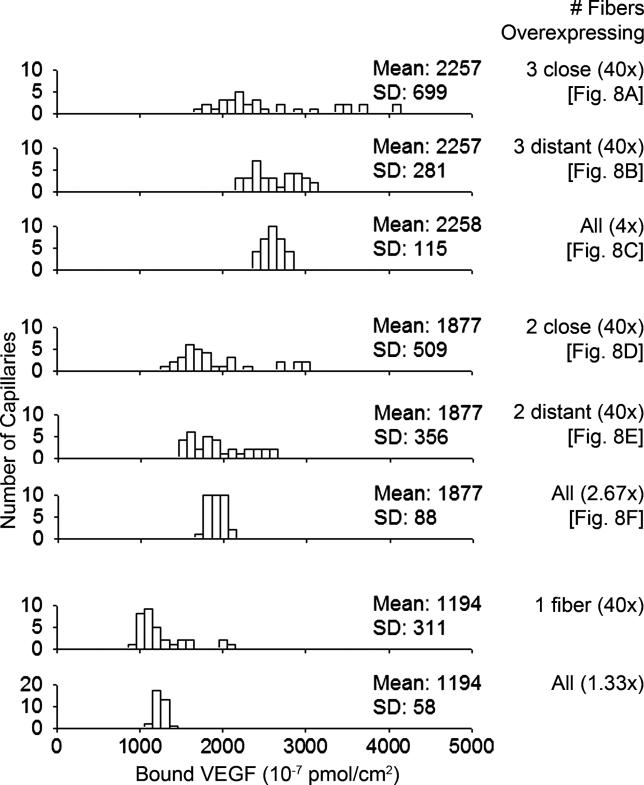
VEGF Binding to Capillaries for Increasing VEGF Dose The distribution of VEGF binding on vessels for one, two, and three 40-fold overexpressing fibers, and the equivalent uniform overexpression. Each tissue is labeled with the number of fibers overexpressing VEGF, the level of overexpression in each fiber, and the panel in Figure 8 for the corresponding VEGF concentration and receptor activation.

To determine the effect of increasing the total VEGF dose delivered to the tissue, we change the density of VEGF-expressing fibers. One, two, or three muscle fibers were each chosen to overexpress VEGF at 40-fold over basal level to study the effect of overexpression density on VEGF distribution (Figures 8 and 9). For overexpression of three adjacent fibers, gradients average 5.6% VEGF/10 μm (36% VEGF/10 μm maximum), and capillaries can bind up to 4,087 10^−7^ pmol/cm^2^ of VEGF (versus 2,049 10^−7^ pmol/cm^2^ for single-fiber overexpression). Choosing distant overexpressing fibers as in Figure 8B and 8E (106 μm between the centers of the fibers) decreases both gradients (average 3.6, maximum 33% VEGF/10 μm) and maximum capillary VEGF binding (3,022 10^−7^ pmol/cm^2^). Decreasing the number of overexpressing fibers to two or one (all at 40-fold over basal expression) does not directly correlate with a decrease in VEGF gradients (5.1% and 4.2% VEGF/10 μm average for two adjacent fibers and single fiber, respectively), but maximum capillary-bound VEGF decreases (2,963 and 2,590 10^−7^ pmol/cm^2^ for two adjacent fibers and single fiber, respectively) due to the decreased total VEGF expression in the tissue.

## Discussion

This study establishes a conceptual framework for the study and prediction of VEGF gradients in tissue, which cannot be measured with present experimental techniques. The effects of VEGF on angiogenesis are widely studied, and experiments have shown that VEGF gradients are necessary for endothelial tip cell migration and guidance during sprouting angiogenesis to prevent malformation of microvasculature [21–23]. Our model predicts that significant gradients (average 3% VEGF/10 μm; greater than 10% in part of the tissue) develop at the scale of the single endothelial cell even under resting conditions. For sprouting vessels, the tip cell is approximately 50 μm long; the gradients are thus sufficient for cell sensing. No study has yet determined whether the endothelial tip cell senses and responds to relative or absolute gradients of VEGF (e.g., in chemotaxis). Our study reports relative gradients in units of percent VEGF/10 μm, but these can be converted to absolute gradients by multiplying the relative value by the mean VEGF concentration in the tissue.

The gradients of VEGF concentration result in significant heterogeneity in the binding of VEGF to VEGF receptors on the capillaries; this may be a reason for the stochasticity of sprout formation—some capillaries become angiogenically active, while others do not. In addition, non-uniform overexpression of VEGF leads to increased gradients and to a different pattern of capillary activation by VEGF compared with uniform overexpression.

Our model is constructed from published rat EDL skeletal muscle data, but parameters such as binding affinity of receptors have never been measured in vivo and others such as free VEGF concentration have never been measured specifically for rat EDL tissue. Receptor expression on endothelial cells has not been measured for rat EDL so our model uses measurements for human skeletal muscle, and we assumed that the same number of receptors per endothelial cell is expressed in human and rat. Our sensitivity analysis of the effect of VEGF receptor concentration on the formation of VEGF gradients predicts that gradients will be smaller if receptor expression is lower in rat than human. Also, we have previously shown that the presence of NRP-1 has a strong effect on VEGF binding kinetics [24], but NRP-1 expression has never been measured in rat skeletal muscle. We predict that VEGF gradients would still be significant in the presence of NRP-1 but the response would depend on the concentrations of VEGFR1 and VEGFR2 in the muscle. Furthermore, an interesting conflict arises regarding the void volume (fraction of interstitial space). Radiotracer data for rat skeletal muscle gives values near 18% [30], while analysis of micrographs reveals much lower values ranging from 5%–10% [11]. The discrepancy remains unresolved, and we used the tracer data for our model.

Our analyses of both VEGF receptor quantity and receptor binding kinetics can also be interpreted as alternative model parameters for different tissues types or demonstrate the response of VEGF gradients to a change in muscle state (e.g., increase in VEGFR2 in response to exercise [48]). Our study shows VEGF gradients in tissue are dependent on VEGF receptor concentrations, and increasing receptor expression on endothelial cells (while keeping binding kinetics constant) results in steeper gradients. By doubling surface expression of VEGFR1 and VEGFR2, maximum gradients nearly double; thus the tissue has the ability to modulate gradients based on cellular expression. Furthermore, recent evidence suggests that skeletal myocytes may also express VEGF receptors, which may have a direct effect on PI3K/Akt signaling (shown to induce the expression of VEGF in both endothelial and non-endothelial cells) [49,50]. The amount of myocyte surface receptor expression has not been quantified and its effect on gradients was not predicted in this study.

Both total and unbound VEGF concentrations have been measured in vivo [10,42], and localization of VEGF has been shown through VEGF staining and microscopy [11]. In our study, we report that the total VEGF content in muscle (free, bound to HSPG, and bound to cell surface receptors) is predicted to be 693 pg/(cm^3^ tissue) under control conditions. This is approximately 0.4% and 2% of experimentally measured whole tissue VEGF concentration for skeletal muscle in human and rat, respectively [3,39]. A portion of remaining VEGF may be distributed within myocytes, and this intracellular content has not yet been quantified in experimental studies. Furthermore, soluble VEGFR1 (sVEGFR1) has not been included in our model, and HSPG concentration in interstitium varies between different skeletal muscle types. Increasing the amount of either HSPG or sVEGFR1 would increase predicted total VEGF in tissue. Our study focuses on VEGF gradients and our results show that neither HSPG concentration nor level of uniform VEGF expression affects the relative gradients.

Currently, there are no measurements of VEGF receptor binding or VEGF distribution in vivo. Our model predicts that approximately half of total VEGF is bound to the cell surface receptors and that less than 3% of VEGF receptors (both VEGFR1 and VEGFR2) are occupied. VEGF in the interstitium is essential to the guidance of capillary sprouts during angiogenesis, and only 2.5% of interstitial (non- cell surface–bound) VEGF is freely diffusible. It is unknown whether HSPG-bound VEGF plays a direct role in guiding capillary sprouts, but studies show that heparin-binding isoforms of VEGF are essential to proper vessel formation [23]. Our model shows that under steady state conditions, relative gradients of free VEGF (measured as a change in free VEGF over distance divided by mean free VEGF) match relative gradients of bound VEGF in the ECM.

The low fractional occupancy of VEGF receptors on endothelial cells indicates that in resting skeletal muscle, approximately 310 molecules of VEGF are bound per cell on average. Endothelial cells may use a system of VEGF signaling amplification to produce angiogenic events. Despite these small quantities of bound VEGF, we have previously shown through Monte Carlo simulations that a continuum model gives valid results for VEGF interactions [26]. The high fraction of unbound receptors in resting skeletal muscle gives the cell the ability to internalize large amounts of VEGF and may provide increased sensitivity to changes in extracellular VEGF concentrations or gradients.

Our sensitivity analysis shows that VEGF gradients are highly sensitive to parameters for VEGF receptor kinetics and concentration values. In our model, HSPG concentration and binding kinetics do not affect relative gradients, and increasing HSPG concentration causes more VEGF to be bound without affecting free VEGF content. Experiments show that HSPG has a significant effect on the mitogenic activity of VEGF [51] and modulates interactions between VEGF and its receptors [52]. Furthermore, HSPG reduces degradation of other signaling molecules such as bFGF [53]. Changing VEGF receptor concentrations (by changing VEGF receptor insertion rate or changing internalization rates), binding affinity, internalization kinetics, or heterogeneity of capillary placement has a significant effect on VEGF gradients. These kinetic rates and concentrations must be better characterized in vivo and in more tissues in order to make more accurate predictions of VEGF gradients.

Our model shows that even under resting physiological conditions, VEGF gradients of up to 12% VEGF/10 μm can exist throughout skeletal muscle. The gradients exist due to the spatial heterogeneity of capillaries within the tissue. When capillaries are uniformly spatially distributed, gradients do not exceed 4% VEGF/10 μm at any location. The model's random capillary placement is typical of capillary distribution in skeletal muscle [32]. VEGF concentrations are predicted to be highest in areas with few capillaries, but deviations in bound VEGF are small between capillaries (mean ±10%). These differences may be significant for capillary sprouting; the threshold in VEGF receptor activation between sprouting and not sprouting is not known. VEGF concentration increases linearly with total secretion, and the concentration of bound VEGF increases linearly for all capillaries.

Our model predicts the angiogenic effect of VEGF using the density of bound VEGF receptors on capillaries as an indication of the probability of capillary proliferation or activation which leads to angiogenesis. Because VEGF receptor binding has low heterogeneity (i.e., low standard deviation) under uniform expression (Figures 2, 7, and 9), most capillaries will be activated to the same degree. That is, if a threshold level of VEGF signaling triggers angiogenic sprouting, most capillaries will exceed (or fall below) the threshold and respond (or not respond) together. For uniform overexpression to stimulate angiogenic sprouting in only a small fraction of the vessels, other distinct intracellular and intercellular signals should ultimately govern where sprout formations occur. It is possible that heterogeneous oxygen distribution under certain conditions, e.g., in exercise, leads to heterogeneity of VEGF secretion via the hypoxia-inducible factor HIF-1α; this scenario should be explored in future studies. For non-uniform overexpression, a subset of capillaries has more VEGF–VEGFR complexes than the others (Figures 7 and 9). Thus, that fraction of the vessels may be activated and initiate angiogenic sprouts while the other vessels do not. For example, for an activation threshold of 2,000 10^−7^ pmol/cm^2^, three vessels will be activated for the case of a single 40-fold overexpressing fiber (Figure 7) while no vessels will be activated in other distributions.

Local overexpression simulations (where only one to three fibers overexpress VEGF) follow experimental observations of transgenic myocyte transplants [19,20]. In those studies, transgenic myocytes overexpress VEGF at levels between 20-fold and 200-fold, which lead to either normal or aberrant (malformed) angiogenesis. The results of our model support experimental observations for both transgenic myoblasts and exercising muscle that VEGF upregulation is necessary for angiogenesis but total dosage of VEGF is insufficient for growth of stable vessels [11,19]. Our model shows that when three adjacent overexpressing VEGF myocytes (at 40-fold expression each) are placed in tissue, surrounding capillaries bind nearly eight times as much VEGF (compared with basal level). The diffusion of VEGF through interstitial space is not fast enough to prevent specific capillaries adjacent to overexpressing myocytes from becoming activated more than others. Therefore, decreasing the number of overexpressing myocytes will not prevent small areas of tissue from developing hemangiomas due to the synergistic activation of capillaries by several adjacent myocytes (in agreement with [19]). Conversely, decreasing the maximum secretion by a single myocyte will decrease the maximum capillary activation independent of the number of adjacent overexpressing myocytes. Furthermore, the activation of specific capillaries (e.g., one-third of all capillaries binding up to 65% more VEGF than other capillaries for a single 40-fold overexpressing fiber; Figure 7) may produce a different pattern of angiogenesis compared with nearly uniform capillary activation resulting from uniform VEGF expression. Because microenvironmental gradients cannot be externally controlled in vivo, in order for transgenic myocytes to be an effective tool for therapeutic angiogenesis, the level of VEGF overexpression may instead be carefully controlled for each myocyte. Although experiments have also shown that myoblasts expressing VEGF_165_ up to 20-fold of basal level can be implanted into heart muscle damaged by myocardial infarction resulting in angiogenesis, improved circulation, and no hemangioma formation [54], the safety of VEGF overexpression therapy is still questioned [55]. Therefore, more experimental studies are needed for a more comprehensive understanding of normal versus aberrant VEGF-induced angiogenesis.

Our model provides a framework for future models to study VEGF gradients, and can be applied to hosts of other physiological and drug-induced conditions (including exercise, ischemia, and VEGF pellet administration), as well as to other tissues (including tumor). The 2-D model can be extended to a 3-D model in order to analyze longitudinal VEGF gradients at the cost of computational complexity. This study also provides a valuable tool to biologists by raising questions concerning both the importance of intracellular VEGF signaling and biophysical changes to the tissue environment when stress is applied to tissue. Many questions still remain unanswered such as whether large quantities of VEGF receptors are expressed on myocytes or on the intraluminal surface of capillaries. These key questions may affect the results we present in this study and provide valuable insight into the complexity of VEGF signaling and interactions with other pro- and anti-angiogenic factors.

In summary, the key findings of this study are: significant VEGF gradients are predicted to exist in tissue in vivo, sensible at the single-cell level; VEGF concentration gradients lead to blood vessel VEGF receptor activation heterogeneity; VEGF gradients increase following VEGF overexpression; and the arrangement of VEGF-overexpressing cells affects VEGF gradients and VEGF receptor activation.

## Materials and Methods

### Numerical solution.

Equations 1–14 were discretized using a fully implicit finite difference algorithm in which first-order spatial and temporal derivatives were expressed with a forward difference scheme and second-order spatial derivatives were expressed with a central difference scheme. The algorithm uses an orthogonal grid with uniform spacing in each spatial coordinate. A grid spacing of 1 μm was used; decreasing the grid spacing to 0.5 μm did not alter the results. Areas of BM are identified by intersections between a grid point in ECM and a grid point within a muscle fiber or capillary. The BM thickness is about one-tenth of the grid size, thus its effect is included in the lumped boundary condition (Equations 3–4 and Equations 5–14). The model geometry implies that a BM must lie between the two grid points and that every BM in the model is discretized into a number of smaller BM areas equal to the total grid intersections passing through every BM space in the model. Discretized BM areas surrounding each capillary or muscle fiber are assumed to be equal in size.

In this study we are interested in the steady state solution. At each simulation step, free VEGF concentration was first obtained using Equations 1–6, and then binding, insertion, and internalization of VEGF receptors at the cell surface and HSPG are solved independently using Equations 7–14. The model iterates until VEGF concentrations reach a steady state. The convergence criterion used was a fractional change less than 10^−5^ for VEGF_120_ and VEGF_164_ at each grid point per step. The model is coded in C++ and requires approximately one hour to reach a steady state solution on a 3.2-Ghz Pentium IV processor with 1 GB of RAM.

## Supporting Information

Figure S1 Total VEGF in Interstitial Space for Uniform Overexpression (172 KB PDF)

Figure S2 VEGF Gradients for Uniform Overexpression (237 KB PDF)

## Abbreviations

BM: basement membrane
EBM: endothelial basement membrane
ECM: extracellular matrix
EDL: extensor digitorum longus
FGF: fibroblast growth factor
HSPG: heparan sulfate proteoglycans
MBM: myocyte basement membrane
MD: myonuclear domain
VEGF: vascular endothelial growth factor
