# Supplementary material for: Computational Model of Vascular Endothelial Growth Factor Spatial Distribution in Muscle and Pro-Angiogenic Cell Therapy
Source: PLoS Comput Biol. 2006 Sep 22;2(9):e127. doi: 10.1371/journal.pcbi.0020127 (PMC1570371; doi:10.1371/journal.pcbi.0020127)
Supplement: Supplementary file 1 [file pcbi.0020127.sg001.pdf]

Supplement to: Computational Model of VEGF Spatial Distribution in Muscle and Pro-Angiogenic Cell Therapy

Feilim Mac Gabhann, James W. Ji, and Aleksander S. Popel

Department of Biomedical Engineering, Johns Hopkins University School of Medicine, Baltimore, MD 21205

Figure S1

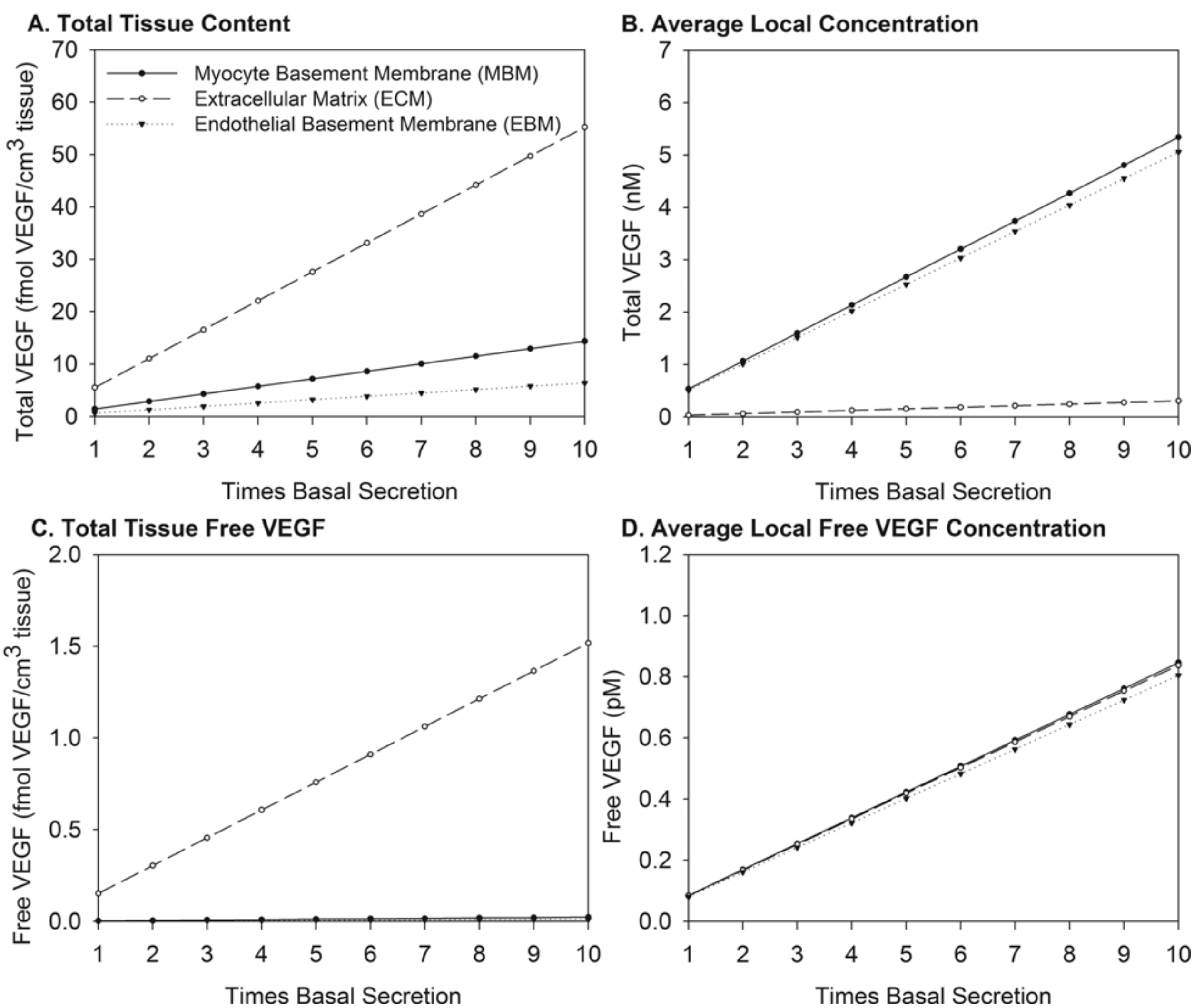

Figure S1. Total VEGF in interstitial space for uniform overexpression. The secretion rate is increased 2 to 10-fold in all fibers uniformly, and total interstitial VEGF concentration increases linearly. Total VEGF includes free $VEGF_{120}$, free $VEGF_{164}$ and HSPG-bound $VEGF_{164}$. **A,** Total VEGF content in the tissue. The MBM, ECM and EBM contain 19%, 73% and 8% of total interstitial VEGF, respectively. Free VEGF comprises 0.2%, 3.4%, and 0.2% of total VEGF in the MBM, ECM and EBM, respectively. **B,** Average local concentrations of total VEGF. VEGF concentrations in the EBM and MBM are 16 and 17 fold higher than in the ECM, respectively, due to the higher concentrations of HSPG in EBM and MBM. **C and D,** Free VEGF concentrations, notations as in (A) and (B). The difference between (A) and (B), and the difference between (C) and (D), are due to the volume of the ECM and basement membranes. The small volume of the EBM and MBM results in a lower tissue concentration of VEGF (A,C) compared to the local concentration (B,D). The difference between (A) and (C), and the difference between (B) and (D), are due to the VEGF binding site densities in the ECM, MBM, and EBM. The higher binding site density in the basement membranes increases the total VEGF in these spaces relative to the free VEGF.
