# Supplementary material for: Computational Model of Vascular Endothelial Growth Factor Spatial Distribution in Muscle and Pro-Angiogenic Cell Therapy
Source: PLoS Comput Biol. 2006 Sep 22;2(9):e127. doi: 10.1371/journal.pcbi.0020127 (PMC1570371; doi:10.1371/journal.pcbi.0020127)
Supplement: Supplementary file 2 [file pcbi.0020127.sg002.pdf]

Supplement to: Computational Model of VEGF Spatial Distribution in Muscle and Pro-Angiogenic Cell Therapy

Feilim Mac Gabhann, James W. Ji, and Aleksander S. Popel

Department of Biomedical Engineering, Johns Hopkins University School of Medicine, Baltimore, MD 21205

Figure S2

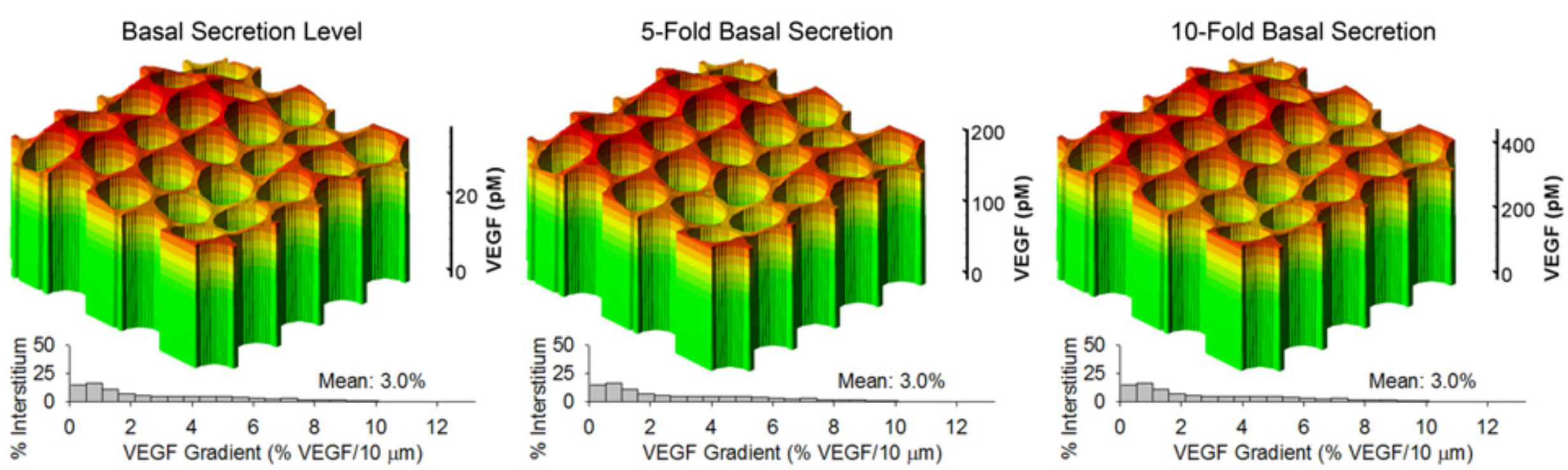

Figure S2. VEGF gradients for uniform overexpression. The average VEGF concentration in the interstitial space increases following overexpression (note the change in scales for concentrations profiles). The spatial distribution does not change, and the relative gradients of VEGF (% VEGF/10 μm) do not change (histograms). Absolute gradients (pM VEGF/10 μm) increase linearly as the average concentration of VEGF in the interstitial space increases. Basal secretion level, 2.7 fmol/(L tissue)/s.
